# Dynamics of competition between coupled spiking networks in the balanced state

**DOI:** 10.1186/1471-2202-16-S1-P211

**Published:** 2015-12-18

**Authors:** Fereshteh Lagzi, Stefan Rotter

**Affiliations:** 1 Bernstein Center Freiburg, Freiburg, Germany; 2 Faculty of Biology, University of Freiburg, Freiburg, Germany

## 

The nonlinear mean-field dynamics of spiking neuronal networks with non-homogeneous connectivity is studied numerically and analyzed mathematically. The network under study is comprised of three subnetworks of either excitatory or inhibitory leaky integrate-and-fire neurons, two of which are of the same type. The excitatory and inhibitory weights are arranged to establish and maintain a balance between excitation and inhibition for a constant external drive. Each subnetwork has random connectivity with fixed in-degree and fixed out-degree for all neurons belonging to a particular population (configuration model). Neurons are also randomly connected with the same probability across different subnetworks; however, depending on their identity, the connection weight is scaled by a common factor. We observed that for a certain regime of ratios of the "within" versus "between" connection weights (bifurcation parameter), network activation spontaneously switches between the two subnetworks of the same identity. In the mean-field model, this phenomenon is explained by a set of coupled stochastic differential equations of Lotka-Volterra type [1,2] that establish competition between the subnetworks. The model shows the same dynamical behavior as observed in simulations of large spiking networks.

The deterministic phase portrait is characterized by two attractors and a saddle node, its stochastic component is essentially given by the multiplicative inherent noise of the system [3]. We observe that the life time distribution of the active states is exponential, therefore, and it appears that noise fluctuations kicks the system from one attractor to the other. The same model for a larger number of populations suggests a general approach to study the dynamics of interacting populations of spiking networks.
